# A new case of radiation-induced breast angiosarcoma

**DOI:** 10.1016/j.ijscr.2019.06.006

**Published:** 2019-06-12

**Authors:** Vincenzo Verdura, Bruno Di Pace, Marina Concilio, Antonio Guastafierro, Gabriella Fiorillo, Luigi Alfano, Giovanni Francesco Nicoletti, Clementina Savastano, Anna Maria Cascone, Corrado Rubino

**Affiliations:** a Multidisciplinary Department of Medical-Surgical and Dental Specialties, Plastic Surgery Unit, Università degli Studi della Campania “Luigi Vanvitelli”, Naples, Italy; b Department of Medicine, Surgery and Dentistry “Scuola Medica Salernitana” PhD School in Translational Medicine of Development and Active Aging, Università degli Studi di Salerno, Salerno, Italy; c Department of Medicine, Surgery and Dentistry, Plastic Surgery Unit, Università degli Studi di Salerno, Salerno, Italy; d A.O.U. San Giovanni di Dio e Ruggi D’Aragona, via San Leonardo, Salerno, Italy; e Plastic Surgery Unit, Department of Surgery, Microsurgery and Medical Sciences, University Hospital Trust, Università degli Studi di Sassari, Sassari, Italy

**Keywords:** Angiosarcoma, Breast-conserving surgery, Radiotherapy

## Abstract

**Introduction:**

Radiation-induced breast angiosarcoma is a severe but rare late complication in the breast-preserving management of breast cancer through surgery and radiotherapy. Often the initial diagnosis is complex given its relatively anodyne nature and the fact that it usually presents in the form of typically multifocal reddish-purple papular skin lesions.

**Presentation of the case:**

We describe the clinical and pathologic findings of a 79-year-old woman, who developed a radiation-induced breast angiosarcoma after around 8 years. She initially refused a mastectomy leading to an adaptation in the management of this cancer.

**Discussion:**

The average latency of secondary angiosarcoma of the breast following radiation therapy is around six years. Breast angiosarcoma is typically considered to affect the dermis, and is therefore cutaneous in origin. An incisional biopsy of the discoloured skin and underlying mass is necessary. The treatment is surgical resection. The role of chemotherapy has not been clearly defined. Most data originate from retrospective case series studies suggesting that angiosarcomas are relatively sensitive to taxanes and anthracyclines.

**Conclusion:**

The preferred treatment is always aggressive surgical removal and, as our atypical clinical case suggests, neoadjuvant chemotherapy in very high doses is also needed. A biopsy of any suspicious breast skin lesion after radiotherapy is recommended. Despite the treatment challenges, our case provides enlightening details on the management of such a rare cancer even when faced with unplanned events which do not always allow for a textbook approach.

## Highlights

• A biopsy of any suspicious breast skin lesion after radiotherapy is recommended.
• Patients with clinical anomalies post-breast cancer surgery and RT need attention.
• The Angiosarcoma was cured due to surgery and high-dose neoadjuvant chemotherapy.
• A correct 6 month follow-up is needed: check-ups, chest X-rays and ultrasound.
• The survival of the patient suggests possible ways to manage this rare tumour type.

## 1 Introduction

Angiosarcomas are highly aggressive and malignant blood vessel tumours originating from endothelial cells which usually develop on the head and neck and can arise spontaneously or in association with many factors [1].

Angiosarcomas that develop in the breast following conservative therapy, namely radiotherapy to treat breast cancer, are exceedingly rare [2]. Due to this low incidence, which is less than 1% [2,3], radiation-associated angiosarcoma (RAAS) represents a diagnostic challenge due to its benign presentation and skin changes that are easily attributed to radiation. Therefore, it is important to take any changes of the breast seriously and to consider RAAS as a differential diagnosis [4].

Surgical resection is rarely curative, and there is only a modest sensitivity of angiosarcoma to taxanes or anthracycline-based chemotherapy [5,6]. However, early detection and radical surgical treatment is potentially curative [7] hence the importance of increased awareness and knowledge regarding the presenting symptoms. This study aims to offer details on the management of the rare tumour in question. This case is reported in line with the SCARE criteria [8].

## 2 Case report

A 79-year-old patient was diagnosed with a right breast carcinoma in October 2007. There was no family history of breast cancer. However, the patient’s medical history indicated: pharmacological treated arterial hypertension, senile arthrosis, contact with the HB virus, thrombocytosis under treatment with Oncocarbide. Moreover, the patient had undergone several surgeries: cholecystectomy, tonsillectomy and in November 2007 received a quadrantectomy of the upper-outer right breast quadrant with radical lymphadenectomy of the right armpit within filtrating ductal carcinoma T3N1G3, Estr. 80%, Pr. Neg, Ki67 > 30%, C-ERB2 negative as a final diagnosis. The patient initially refused a mastectomy because of its mutilating approach. She underwent chemotherapy and radiotherapy succeeded by oncological follow-up which consisted in clinical examinations every 6 months, a chest X-ray, a bilateral ultrasound check of the breast and axilla, an abdominal ultrasound check, a bone scintigraphy and dosing of tumour markers (αFP, CEA, Ca125, Ca 15.3, TPA). There were no relevant data until 2014. From September 2015 the patient reported a swelling of the right breast with progressive and probable appearance of neoplastic recurrence that progressively underwent ulceration from the outer quadrant and to the whole breast.

The patient underwent an MRI test of both breasts with and without contrast that reported that at the junction of the upper quadrants and in the lower outer quadrant of the right breast there were oval formations with a maximum size of 16 × 20 mm with early enhancement after the injection of intravenous contrast and at the junction of the outer quadrants there was also a coarse formation with the maximum size of 4 × 3.5 mm which reached the cutaneous layer and showed widespread enhancement after the injection of intravenous contrast. On the cutaneous profile, oval formations were noted as infiltration.

In May 2016, a fine-needle aspiration cytology (FNAC) of the breast mass on the right with multiple skin nodules was carried out and results revealed malignant cells. The cytological test showed blood and a few ductal cells with a definite nuclear atypia corresponding to category C5 according to European guidelines. This was followed by analysing the receptors and the proliferating fraction which showed that the estrogenic receptor (clone SP1) and Ki67 were not evaluable. Furthermore, the scarcity of the diagnostic cells did not allow for an evaluation of the receptors and a targeted biopsy was prescribed.

However, the patient was reluctant to have the targeted biopsy after receiving the feedback about the malignant cells and indeed was terrified that she may have had advanced stage cancer. In light of this indecision and considering the amount of time which had passed for the likelihood of successful surgical radicality, the decision was made for neoadjuvant chemotherapy to be administered.

In June 2016, the patient started 8 cycles of chemotherapy according to the Carboplatin-Gemcitabine protocol and in August 2016 there was a clear improvement in the local conditions of the right breast.

Therefore, in September 2016, the patient was hospitalised: the neoplasm appeared to be ulcerated with a fungus appearance in particular in the outer quadrant, with some necrotic features of chemotherapy. There was no palpable tumefaction in the surrounding lymphatic areas.

During the preoperative check-up, the patient underwent a pre-anaesthetic evaluation and a cardiological consultation. Lab tests, ECG and thoracic X-ray(AP; LL) were deemed necessary: the last test showed a thickened aspect of the soft tissues in the right breast region.

Therefore, a monolateral right mastectomy surgery was then planned and carried out with a primary surgical wound closure despite the ulceration and fungation. The mastectomy tissue sample measured a total of 15 × 12 × 6 cm and was lined with skin without the nipple; the skin surface had a vegetative mass of 60 mm and was ulcerated with extensive subcutaneous haemorrhage and a further two haemorrhagic masses both of 40 mm. No complications occurred. The patient’s general conditions were good and four days later, the patient was discharged, and clinical check-ups were planned.

The microscopic examination in the anatomic pathology report showed malignant neoplastic proliferation with malignant endothelial cells and large haemorrhagic areas. There was obvious mitosis with the presence of positive neoplastic cells for VIM, CD31, CD34 and negativity for broad-spectrum CK, CA, ER, PR, c-erb-2, EGFR, Ki67: 60% in 3 fields (40×). It was identified as a high-grade angiosarcoma. During the mastectomy, a pectoralis major muscle biopsy of 1.4 × 3.5 × 1 cm was also performed, and the histological diagnosis was tumour-free fibromuscular tissue. After 1 year of treatment the patient was in good general condition and underwent regular follow-ups.

## 3 Discussion

Most angiosarcomas develop in the skin or superficial soft tissue, while only 20% are in the deep soft tissues. The incidence of radiation-induced angiosarcoma ranged from 0.09 to 0.16% [9,10], despite an increasing number of case reports showing increasing secondary angiosarcomas following radiation therapy [11].

Secondary angiosarcomas are found in older women with a history of breast conservation therapy and radiation therapy since breast carcinoma commonly occurs in women between the ages of 55 and 69 [12]. In contrast, primary angiosarcomas account for 0.04% of all malignant breast tumours [13], 1 in 2.000 of primary breast cancers. These occur in young women in their thirties and forties [12].

The average latency of secondary angiosarcoma of the breast following radiation therapy is around six years, with some studies reporting the occurrence of angiosarcoma as soon as 1–2 years after radiation and as late as 41 years after radiation [14,15]. Overall, the occurrence of breast angiosarcoma post-radiation appears to be shorter compared to radiation induced sarcomas in general, which typically have a latency period of 10–12 years [15]. Our case is placed in the middle of the aforementioned time estimates.

Breast angiosarcoma is typically seen as affecting the dermis within the radiation field making these angiosarcomas cutaneous in origin [9,14], moreover, the association between radiation and angiosarcoma of the breast has been reported in literature [16]. Many cases of angiosarcoma developing in the breast parenchyma, arising from parenchymal vascular endothelial cells, have also been reported [10,13]. There are proposed mechanisms for the development of radiation-induced angiosarcoma link lymphedema as a causative factor, such as chronic lymphedema [16].

**Fig. 1:**
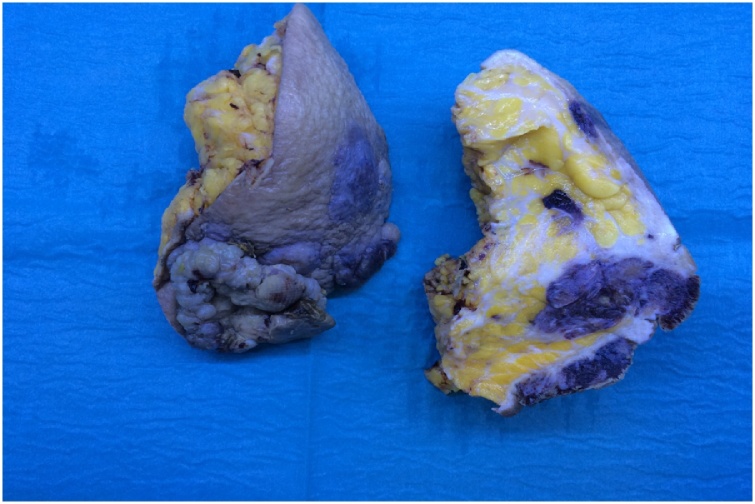
Angiosarcoma, gross appearance: mammary mass with poorly defined margins with hemorrhagic foci.

An incisional biopsy of the discoloured skin and underlying mass is the most accurate and fastest way to obtain a diagnosis [12] as well as FNAC even if, as in our case, the sample was not sufficient for an adequate and complete diagnosis. The macroscopic aspect of the tumour was of a white-greyish exophytic mass with ill-defined margins. The cutting surface showed the presence of multiple hemorrhagic areas (Fig. 1).

**Fig. 2:**
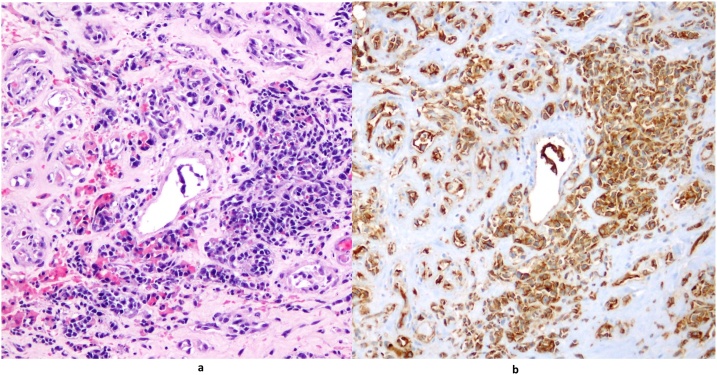
a) (E/E 20x): angiosarcoma, intermediate grade. Irregular sheets of small spindle cells with cellular nodules. b) (CD31 20x): immunohistochemistry for CD31 decorating endothelial cells of angiosarcoma.

**Fig. 3:**
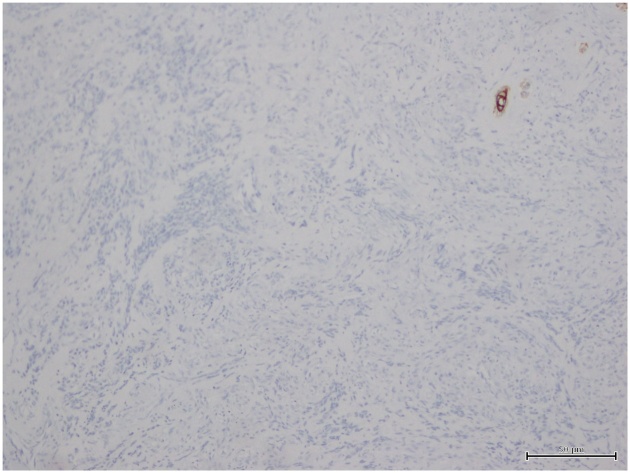
(CK 10x): negative Immunoreactivity for Cytokeratin (CK) is shown in angiosarcoma.

The microscope revealed irregular anastomosing vascular channels lined by one or more layers of endothelial cells that showed hyperchromatic and enlarged nuclei arranged to form nodules composed of spindle cells with swirling patterns (Fig. 2a). Immunohistochemistry showed reactivity for CD31 (Fig. 2b) and negativity for CK which is useful for distinguishing the angiosarcoma from the carcinoma (Fig. 3) [17,18,19]. In fact, typical immunohistochemical expression profiles for angiosarcomas include upregulation of certain vascular-specific thyroxin kinase receptors, including, *TIE1-2* and *VEGFR1*, *VEGFR2* and downregulation of *VEGFR* ligand expression [20,21].

The treatment for angiosarcomas is surgical resection with a mastectomy that aims to obtain negative margins [22]. Prognosis has been historically poor especially for radiation-induced and large deep soft tissue angiosarcomas with a median survival of 25 months [23].

The role of chemotherapy has not been clearly defined. Most data originate from retrospective case series studies or even case reports suggesting that angiosarcomas are relatively sensitive to taxanes and anthracyclines with an initial overall response rate from 20% to 60% [24].

## 4 Conclusion

Overall, although RAASs are rare tumours, a higher incidence of this type of tumour can be expected in the future due to the increasing popularity of breast-conserving surgery of malignant glandular tissue breast tumours followed by mandatory high dose radiation therapy.

The RAAS was eradicated thanks to the surgical treatment (simple mastectomy) and the neoadjuvant chemotherapy with a correct follow-up. Clinical examinations, chest X-rays, bilateral ultrasound checks of the breast and axilla and abdominal ultrasound checks every 6 months are necessary, in addition to bone scintigraphy and dosing of tumour markers as indicated by the oncologist’s timings.

A high level of suspicion should be exercised when patients who receive breast conservation surgery and radiation present with any type of skin discoloration or suspected swellings. Therefore, a biopsy of any suspicious breast skin lesion after radiotherapy is recommended, even in cases where the radiological evaluation does not concur with the physical examination [25]. The preferred treatment is always aggressive surgical removal and, as our atypical clinical case suggests, neoadjuvant chemotherapy in very high doses is also needed.

Despite the treatment challenges, our case provides enlightening details on the management of such a rare cancer within standard clinical and surgical practice. The survival of the patient suggests possible ways in which diagnosis and treatment can be performed for this type of rare tumour even when faced with unplanned events which do not always allow for a textbook approach.

## Declaration of Competing Interest

There are no conflicts of interest to declare.

## Sources of funding

There were no sources of funding to be acknowledged.

## Ethical approval

The study is exempt from ethical approval as the procedure performed is in line with current guidelines.

## Consent

Written informed consent was obtained from the patient for publication of this case report and accompanying images. A copy of the written consent is available for review by the Editor-in-Chief of this journal on request.

## Author contribution

Vincenzo Verdura: paper writer, study concept.

Bruno Di Pace: paper writer, study concept.

Marina Concilio: data collection.

Antonio Guastafierro: data collection.

Gabriella Fiorillo: data collection.

Luigi Alfano: surgical operator of the patient.

Giovanni Francesco Nicoletti: study concept.

Clementina Savastano: oncology therapist.

Anna Maria Cascone: pathology report.

Corrado Rubino: study concept.

## Registration of research studies

N/A.

## Guarantor

Vincenzo Verdura.

## Provenance and peer review

Not commissioned, externally peer-reviewed.
